# Deep Proteomic Analysis Reveals Intermuscular Variation Associated with Chicken Meat Quality

**DOI:** 10.3390/foods15214010

**Published:** 2026-11-09

**Authors:** Yanju Shan, Jingting Shu, Xiaojun Ju, Ming Zhang, Yifan Liu, Gaige Ji, Yunjie Tu, Yongli Wang

**Affiliations:** Jiangsu Institute of Poultry Science, Yangzhou 225125, China; shanyanjusyj@163.com (Y.S.);

**Keywords:** broiler, muscle fiber type, meat quality, quantitative proteomics, postmortem metabolism

## Abstract

To elucidate the molecular mechanisms underlying chicken meat quality, this study first evaluated variation in chicken meat quality with respect to age, breed, sex, and muscle location. High-throughput quantitative proteomic analysis was then performed on the pectoralis major muscle (PEM) and medial gastrocnemius muscle (MGM), a leg muscle subpopulation previously shown to be enriched in slow-twitch oxidative fibers. Marked differences were observed between the PEM and leg muscle in pH, meat color, tenderness, water-holding capacity (WHC), amino acid composition, intramuscular fat composition, and intramuscular fat content. A total of 4413 proteins were identified in PEM and MGM (*n* = 3 biological replicates per muscle type), of which 516 were differentially abundant. Functional enrichment analyses of protein domains, GO terms, and KEGG pathways for the differentially abundant proteins indicated that the intermuscular proteomic differences were mainly associated with carbohydrate metabolism, purine metabolism, calcium signaling, and proteostasis. Protein–protein interaction (PPI) analysis further revealed that the differential expression of node proteins, including GAPDH, TPI1, PGM1, LDHA, ENO3, FHL1, NT5E, RYR2, and MRPS6, may be associated with intermuscular meat quality variation in the early postmortem period. These findings revealed molecular disparities between chicken skeletal muscles with distinct phenotypes and may facilitate postmortem quality control in the poultry meat industry.

## 1. Introduction

Meat quality is a decisive determinant of consumer acceptance and the economic sustainability of the broiler industry. Despite substantial genetic gains in growth rate and feed efficiency, the sensory and technological quality of broiler meat has often declined as production efficiency increases [1,2]. This trade-off has created a pressing challenge for the broiler industry, as production efficiency must be reconciled with the quality attributes that consumers demand [3,4]. Muscle fibers, the fundamental structural and metabolic units of skeletal muscle, are primary determinants of meat quality. Their compositional variation across breeds and anatomical locations shapes the postmortem biochemical changes in muscle and profoundly influences multiple quality traits, including WHC, color, tenderness, and processing suitability [5,6]. Fiber-type characteristics are established during embryogenesis and subsequently modulated by neural, endocrine, and mechanical inputs throughout development [7,8,9]. Within skeletal muscle, the distribution of fiber types is governed by an intricate regulatory network involving numerous key regulators and signaling pathways. Notably, the mechanisms controlling muscle fiber composition can differ across animal breeds and species [10].

Proteins are the fundamental executors of cellular functions, bridging the gap between genotype and phenotype [11]. In skeletal muscle, proteins are key determinants of fiber-type specification, and variations in their composition and function profoundly influence meat quality traits [12]. Proteomics, by providing a global view of protein expression in an organism, has therefore become a pivotal tool for elucidating the molecular basis of meat quality and identifying potential regulatory targets [13,14,15]. The in-depth elucidation of protein function largely depends on the precise detection of low-abundance proteins, and recent advances in mass spectrometry have greatly propelled this field. The Orbitrap Astral mass spectrometer, which integrates high-resolution Orbitrap detection with ultra-fast Astral mass analysis, has markedly improved the robust identification and quantification of low-abundance proteins [16]. Proteome investigations in skeletal muscle of mammals, including pigs [17], cattle [18], and sheep [19], have uncovered several signaling pathways and metabolic networks that may govern fiber-type specification and meat quality. However, whether these regulatory networks are functionally conserved in chickens remains to be determined.

In China, white-feathered broilers, meat-type crossbred chickens, and yellow-feathered broilers occupy distinct market niches shaped by their growth performance and meat quality traits. Here, we selected Arbor Acres broilers (AAB), 817 crossbred chickens (817C), and Wenchang chickens (WCC) as representatives of these three genetic groups and comprehensively assessed the effects of breed, sex, and age on meat quality traits in both the pectoralis major muscle (PEM) and leg muscle (LM). Furthermore, using Orbitrap Astral mass spectrometry, we performed global proteomic profiling of the two muscle types to decipher their distinct molecular signatures. Our findings may deepen the current understanding of the molecular basis underlying intermuscular variation in meat quality and provide a valuable resource for improving broiler meat quality and understanding muscle biology.

## 2. Materials and Methods

### 2.1. Ethics Statement

The animal study was approved by the Animal Care and Use Committee of the Jiangsu Institute of Poultry Science (approval no. 20250317, approved on 17 March 2025). All animal experiments used in this study were conducted in accordance with the Guide for the Care and Use of Laboratory Animals by the Ministry of Science and Technology of China (Beijing, China). Every effort was made to minimize animal suffering.

### 2.2. Sample Collection

The overall experimental design, including breed, age, sex, and muscle samples, is summarized in Table 1. Market-ready 817C (56 d) and WCC (120 d) were sourced from commercial markets and the experimental poultry farm of Jiangsu Institute of Poultry Science, respectively. One-day-old male AAB chicks were sourced from an authorized supplier in China, randomly allocated to experimental groups, and raised under standardized conditions until 49 d. Before slaughter, all birds were fasted for 24 h, with no access to water for the last 2 h, and then humanely exsanguinated by carotid artery severing. Immediately after dissection, skeletal muscle samples, including the PEM, LM, and medial gastrocnemius muscle (MGM, an LM subpopulation previously shown to be enriched in slow-twitch oxidative fibers), were excised. Because meat quality analysis requires a sufficient amount of muscle tissue, the LM rather than MGM was selected for meat quality assessment. For the age effect on edible meat quality, 40 AAB were used (10 per age group), with 10 biological replicates per age group. For the sex effect on edible meat quality, 20 817C were used (10 per sex group), with 10 biological replicates per sex group. For the breed effect on meat nutritional quality, 34 male chickens at market age were used (AAB, *n* = 10; 817C, *n* = 9; WCC, *n* = 15), with 10, 9, and 15 biological replicates, respectively. For proteomic analysis, PEM and MGM samples were collected from three 120 d WCCs randomly selected from the 15 male WCCs used in the meat quality experiment, yielding three biological replicates per muscle type and six samples in total. No samples were pooled. The individual bird was used as the experimental unit. Samples for meat quality assessment were stored at 4 °C, whereas those intended for quantitative proteomics analysis were snap-frozen in liquid nitrogen and preserved at −80 °C until further processing.

### 2.3. Meat Quality Analysis

The edible quality of meat, including pH, color, tenderness, and water loss rate, was determined using conventional methods at 24 h postmortem. Subcutaneous fat was removed from the PEM and LM before analysis. All measurements were performed at room temperature unless otherwise stated, and the mean of technical replicates was used as the representative value for each biological sample. The pH was measured using a pH meter (pH-STAR, Matthaus, Eckelsheim, Germany) calibrated with standard buffers (pH 4.00, 6.86, and 9.18), with the electrode inserted approximately 0.5–1 cm into the muscle sample. Three distinct positions per sample were measured. Color was measured using a colorimeter (NR20XE, 3nh, Shenzhen, China) calibrated with standard white and black ceramic tiles, and the lightness (L*), red–green axis (a*), and yellow–blue axis (b*) values were recorded. Tenderness was evaluated by measuring shear force on raw meat. After removal of visible connective tissue, muscle blocks (1 cm thick × 1 cm wide), oriented parallel to myofiber alignment, were sheared perpendicularly to the myofiber orientation in triplicate using a meat shearing apparatus (C-LM4, Tenovo, Beijing, China). The water loss rate was measured using a strain-controlled unconfined pressure apparatus (YYW-2, NSIF, Nanjing, China). First, approximately 0.5 g of the meat sample was accurately weighed (W_1_). The sample was then placed between medical gauze and filter paper and subjected to a compressive load of 35 kg for 5 min. After the applied force was removed, the sample was reweighed (W_2_). Finally, the water loss rate was calculated using the following formula: water loss rate (%) = (W_1_ − W_2_)/W_1_ × 100%. A higher water loss rate indicates lower water-holding capacity (WHC).

To systematically evaluate the nutritional quality of the muscle samples, samples were minced and homogenized, and the contents of key nutritional components were determined. Specifically, amino acid (AA) content in the muscle sample was determined using high-performance liquid chromatography (HPLC, Agilent 1260, Santa Clara, CA, USA) following hydrochloric acid hydrolysis based on the method of Heinrikson and Meredith [20]. Briefly, a 0.1 g ground muscle sample was hydrolyzed with 20 mL of 6 M hydrochloric acid at 110 °C for 22 h. After cooling, the hydrolysate was diluted to 25 mL with deionized water. An aliquot of 100 μL was dried under vacuum at 60 °C for 2 h. The residue was protected under nitrogen gas and derivatized with 50 μL of freshly prepared reagent (ethanol–phenyl isothiocyanate–water–triethylamine = 7:1:1:1, *v*/*v*) at room temperature for 30 min. Following derivatization, the mixture was reconstituted in an acetonitrile-based mobile phase, vortexed, filtered through a 0.45 μm organic membrane, and analyzed by HPLC. The Soxhlet extraction method was used to determine the intramuscular fat (IMF) content in the muscle sample according to GB 5009.6 [21]. Fatty acid (FA) content in the muscle sample was determined using gas chromatography (GC, Agilent 7890A, Santa Clara, CA, USA) coupled with an acid hydrolysis technique, based on the hydrolytic extraction method of GB 5009.168 [22].

### 2.4. Protein Extraction and Digestion

Total proteins were extracted from PEM and MGM samples (*n* = 3 per group) using a high-intensity ultrasonic processor (SCIENTZ-IID, Scientz, Ningbo, China) in lysis buffer containing 1% Triton X-100 and 1% protease inhibitor cocktail. The protein concentration was determined with a BCA kit (Beyotime, Shanghai, China) according to the manufacturer’s instructions. Proteins were precipitated with trichloroacetic acid and washed with pre-chilled acetone. After air-drying, proteins were redissolved in 200 mM triethylammonium bicarbonate buffer and sonicated. Trypsin was added at an enzyme-to-protein mass ratio of 1:50 for overnight digestion at 37 °C. The samples were reduced with 5 mM dithiothreitol for 30 min at 56 °C, followed by alkylation with 11 mM iodoacetamide for 15 min at room temperature in the dark. Subsequently, additional trypsin was added at a trypsin-to-protein mass ratio of 1:100, and digestion was continued for an additional 4 h. The digested peptides were finally purified by desalting using a Strata X SPE column (Phenomenex, Torrance, CA, USA).

### 2.5. LC-MS/MS Analysis

The mobile phase consisted of solvent A (0.1% formic acid in water) and solvent B (0.1% formic acid in 80% acetonitrile/water). The tryptic peptides (400 ng) were dissolved in mobile phase A and directly loaded onto a home-made analytical column with an integrated spray tip (100 μm i.d. × 15 cm) packed with 1.9 μm/120 Å ReproSil-Pur C18 resins (Dr. Maisch GmbH, Ammerbuch, Germany). Peptides were separated on a Vanquish Neo ultra-high-performance liquid chromatography (UHPLC) system (Thermo Scientific, Waltham, MA, USA) at a constant flow rate of 400 nL/min using solvent A and solvent B. The gradient program was as follows: 0–0.5 min, 4% B; 0.5–0.6 min, 4–8% B; 0.6–13.6 min, 8–22.5% B; 13.6–20.5 min, 22.5–35% B; 20.5–20.9 min, 35–55% B; 20.9–21.4 min, 55–99% B; 21.4–22.6 min, 99% B. The separated peptides were ionized via a nanoelectrospray ion source at 1900 V and analyzed on an Orbitrap Astral mass spectrometer (Thermo Scientific, Waltham, MA, USA). Precursors were analyzed in the Orbitrap detector, and fragments were analyzed in the Astral detector. The full MS scan was acquired at a resolution of 240,000 over a scan range of 380–980 *m*/*z*. The MS/MS scan was fixed at a first mass of 150.0 *m*/*z* at a resolution of 80,000. Data were acquired in data-independent acquisition (DIA) mode using 300 consecutive 2 *m*/*z* isolation windows with no overlap, spanning 380–980 *m*/*z*, at a cycle time of 0.6 s. Peptide ions were fragmented in the high-energy collisional dissociation (HCD) cell at a normalized collision energy of 25%. The automatic gain control target was set to 500%, with a maximum injection time of 3 ms.

### 2.6. Proteomic Data Processing and Functional Annotation

The DIA data were processed using the Pulsar search engine embedded in Spectronaut (v.18) software with default parameters. Tandem mass spectra were searched against the Gallus_gallus_9031_PR_20240807.fasta (43,711 entries) concatenated with a reverse decoy database. Trypsin/P was specified as the cleavage enzyme, allowing up to 2 missed cleavages. Carbamidomethylation on cysteine was set as a fixed modification, and acetylation on the protein’s N-terminus and oxidation on Met were set as variable modifications. The false discovery rate (FDR) for protein, peptide, and peptide-spectrum match identifications was adjusted to <1%. To obtain high-quality analysis results, the database search results were further filtered, and identified proteins were required to contain at least one peptide. A protein was considered detected in a given muscle type if it was identified in at least one biological replicate. Peptides with zero abundance were replaced with missing values, which were left untreated. For protein quantification, the raw LC-MS datasets were converted into matrices of normalized protein intensity (I), which were further transformed into a relative quantitative value (R) by centralization using Rij = Iij/Mean(Ij), where i and j represent the sample and protein, respectively. Fold change (FC) was calculated as the ratio of the mean abundance in MGM to that in PEM. Log2-transformed quantification values were compared using a *t*-test. Proteins with a *p*-value < 0.05 and |log2FC| > 0.585 were identified as differentially abundant proteins (DAPs).

DAPs were functionally annotated using several databases. Eukaryotic Orthologous Group (KOG) annotation was performed using the EggNOG database; protein domain annotation was carried out using PfamScan (v1.6) based on the Pfam database, and subcellular localization was predicted using the WoLF PSORT software (v0.2). Functional enrichment analysis of Gene Ontology (GO) terms, the Kyoto Encyclopedia of Genes and Genomes (KEGG) pathways, and protein domains was performed using Fisher’s exact test, with all identified proteins as the background set. Enrichment was considered significant when BH-adjusted *p* (q) < 0.05 and fold enrichment > 1.5. Furthermore, hierarchical clustering analysis based on protein functional enrichment was performed for DAPs grouped by different fold changes to explore potential associations and differences in specific functions. A protein–protein interaction (PPI) network was constructed using the STRING database (https://cn.string-db.org/; accessed on 8 July 2026), and only interactions with a confidence score > 0.8 were retained for downstream analysis. The PPI network was visualized using Cytoscape 3.10.3.

### 2.7. Statistical Analysis

All data from wet-lab experiments were analyzed using SPSS software (version 27.0) and are presented as mean ± standard error of the mean (SEM). The experimental unit was the individual bird. For comparisons between two tissues from the same bird, we used a paired-samples *t*-test, and significance was indicated by asterisks (*: *p* < 0.05; **: *p* < 0.01; ***: *p* < 0.001). For multiple group comparisons, one-way ANOVA was performed, followed by Tukey’s HSD test. A *p*-value < 0.05 was considered statistically significant, denoted by different lowercase superscripts. For Figures 2 and 3, WorkBuddy v5.3.13 was used solely to assist with the visualization and layout of the data and 3. All data and scientific content were generated by the authors.

## 3. Results

### 3.1. Significant Difference in Meat Quality Between PEM and LM

Our prior study has revealed significant differences in muscle fiber type composition between the PEM and LM in chickens [23]. To further evaluate whether these structural variations translate into distinct meat quality profiles, we evaluated the edible quality of these two muscle types, including pH, color, tenderness, and water loss rate, in two commercial broiler breeds (AAB and 817C). In AAB, we assessed the effect of age on these meat quality parameters in both the PEM and LM. The results showed that while the pH of the PEM remained stable from 28 to 49 d of age, LM pH increased progressively and was significantly higher than that of the PEM at all time points examined (35 d: *p* < 0.05; 42 d: *p* < 0.01; 28 d and 49 d: *p* < 0.001; Figure 1A). Meat color analysis revealed that at 28 d and 35 d, LM maintained higher L*, a*, and b* values than PEM. However, by 49 d, this pattern reversed across all three parameters, with PEM exhibiting higher values than LM (Figure 1B). As expected, LM exhibited greater toughness than PEM, as evidenced by significantly higher shear force values at all tested time points (28 d and 49 d: *p* < 0.001; 35 d: *p* < 0.05; 42 d: *p* < 0.01; Figure 1C). Water loss rate, used to assess WHC, was consistently higher in PEM than in LM, and significant differences were observed at 28 d and 49 d (*p* < 0.05; Figure 1D). In 817C at market age (56 d), sex did not affect the patterns of differences in these meat quality parameters between the PEM and LM, which were consistent with those observed in AAB. Specifically, in both sexes, LM exhibited higher pH (*p* < 0.001; Figure 1E) and shear force (Figure 1G) but lower water loss rate (roosters: *p* < 0.001; hens: *p* < 0.01; Figure 1H) than PEM. Regarding meat color, there were no significant differences between PEM and LM within either sex for L*, a*, or b* (*p* > 0.05; Figure 1F).

To expand our comparative analysis, we further evaluated the nutritional quality (IMF, AA, and FA content) of the PEM and LM from three breeds (AAB, 817C, and WCC) at their respective market age. Remarkably, the overall pattern of these meat quality traits was highly consistent between PEM and LM across all three chicken breeds (Figure 2 and Figure 3). Specifically, compared with PEM, LM exhibited significantly higher IMF content and lower total AA content across all three chicken breeds (*p* < 0.001). However, when normalized to total AA content, LM showed higher proportions of umami AA and aromatic AA than PEM (Figure 2). Regarding FA composition, the total FA content was significantly higher in LM than in PEM across all three chicken breeds (Figure 3; *p* < 0.001). Notably, LM exhibited significantly higher absolute and relative contents of total unsaturated fatty acids (UFA) than PEM (Figure 3; *p* < 0.01–0.001). This increase was particularly pronounced for total monounsaturated fatty acids (MUFA), with significantly higher absolute and relative contents in LM than in PEM (Figure 3; *p* < 0.05–0.001). Compared among the three breeds, WCC exhibited significantly higher total AA content in both PEM and LM than the other two breeds (*p* < 0.05), as well as higher absolute and relative contents of sweet AA and total MUFA (Figure 2 and Figure 3). These results indicate that the marked differences in meat quality between PEM and LM were observed in the respective age, sex, and breed comparisons. These differences may be associated with distinct muscle fiber type composition inherent in the two muscles.

### 3.2. Proteomic Profiles Reveal Distinct Molecular Signatures Between PEM and MGM

To elucidate the molecular basis of meat quality divergence linked to muscle fiber type composition, we used LC-MS/MS-based proteomics to compare protein expression profiles between PEM (predominantly fast-twitch glycolytic fibers) and MGM (an LM subpopulation enriched in slow-twitch oxidative fibers) [23]. Following database searching, high-confidence identifications were obtained by applying stringent filters against low-confidence and non-specific peptide-spectrum matches. In total, 22,223 unique peptides were identified, mapping to 4413 proteins in PEM and MGM (Table 2). Of these, 4335 (98.23%) proteins were shared in two tissues and used for subsequent quantitative analyses. A small subset of proteins was detected only under the present analytical conditions: In total, 5 proteins (0.11%) were uniquely detected in PEM, whereas 73 proteins (1.65%) were exclusive to MGM (Figure 4A). The peptide length distribution predominantly ranged from 7 to 20 amino acids, consistent with the expected patterns of tryptic digestion and optimal fragmentation efficiency for MS analysis (Figure 4B). The majority of identified proteins (85.81%) contained two or more peptides (Figure 4C). These proteins exhibited a wide molecular weight distribution, predominantly in the 10–140 kDa range (Figure 4D). Proteomic profiling by principal component analysis (PCA) revealed a pronounced separation between PEM and MGM groups, primarily along PC1, which accounted for 45.10% of the total variance (Figure 4E).

A total of 516 DAPs were identified between PEM and MGM. Among these, 327 proteins were more abundant in MGM, whereas 189 proteins were less abundant (Figure 5A; Supplementary Table S1). Hierarchical clustering revealed that these DAPs clearly segregated into two distinct clusters (Figure 5B). PCA based on DAPs still yielded robust discrimination between the two muscle tissues (Figure 5C). Given that protein function is closely associated with subcellular localization, we predicted the subcellular localizations of these DAPs. The results indicated that among all DAPs, 180 proteins (34.88%) were assigned to the cytoplasm, 140 proteins (27.13%) to the nucleus, and 66 proteins (12.79%) to the mitochondria (Figure 5D). Notably, the more abundant and less abundant DAPs displayed differential subcellular distribution patterns. Specifically, DAPs of the two groups were predominantly localized in the cytoplasm and nucleus; however, less abundant proteins exhibited relatively higher proportions in the cytoplasm (46.03% vs. 28.44%) and mitochondria (16.93% vs. 10.40%), whereas more abundant DAPs showed relatively higher proportions in the nucleus (31.80% vs. 19.05%) and plasma membrane (16.21% vs. 3.70%) (Figure 5E). Furthermore, KOG annotation revealed that more abundant DAPs were distributed across all 24 categories, whereas less abundant DAPs were present in 22 categories (Figure 5F). Among the more abundant DAPs, the category [T] signal transduction mechanisms was the most abundant, while the category [G] carbohydrate transport and metabolism was the most prevalent among the less abundant DAPs. Interestingly, three categories, including [O] posttranslational modification, protein turnover, chaperones, [R] general function prediction only, and [T] signal transduction mechanisms, were highly represented in both more abundant and less abundant DAP groups, with each ranking among the top four in both groups. Collectively, these findings suggest that the distinct proteomic profiles of PEM and MGM serve as a molecular link between structural diversity and meat quality attributes.

### 3.3. Functional Analyses of DAPs Identified Between PEM and MGM

To investigate the functional characteristics of DAPs identified between PEM and MGM, we performed enrichment analyses for protein domains, GO terms, and KEGG pathways. Protein domain enrichment analysis identified 8 significantly enriched domains, including the phosphorylase b kinase C-terminal domain (PF19292), LIM domain (PF00412), and fructose-bisphosphate aldolase class-I (PF00274) (*q* < 0.05; Figure 6A; Supplementary Table S2). We classified 30 significantly enriched GO terms into three categories: biological process (BP), cellular component (CC), and molecular function (MF) (*q* < 0.05; Figure 6B; Supplementary Table S3). The top enriched terms in BP were glucose catabolic process (GO:0006007), NAD biosynthetic process (GO:0009435), and pyridine nucleotide biosynthetic process (GO:0019363), while the top CC terms were myofibril (GO:0030016), sarcomere (GO:0030017), and contractile fiber (GO:0043292), and the top MF terms were calmodulin-dependent protein kinase activity (GO:0004683), structural constituent of muscle (GO:0008307), and cytoskeletal protein binding (GO:0008092). Notably, KEGG pathway analysis revealed significant enrichment of pathways related to skeletal muscle glucose metabolism and fiber type transformation, including glycolysis/gluconeogenesis (map00010), pentose phosphate pathway (map00030), glucagon signaling pathway (map04922), and calcium signaling pathway (map04020) (*q* < 0.05; Figure 6C; Supplementary Table S4).

To examine functional differences among DAPs with distinct expression patterns, we categorized them into four groups (Q1–Q4) based on FC values. Q1 and Q2 proteins were less abundant in MGM (vs. PEM), whereas Q3 and Q4 proteins were more abundant (Figure 7A). KEGG pathway and protein domain enrichment analyses were then performed for each group. KEGG pathway analysis showed that Q1 proteins were significantly enriched in pathways related to carbohydrate metabolism, including glycolysis/gluconeogenesis, the pentose phosphate pathway, and the glucagon signaling pathway (Figure 7B). In contrast, Q4 proteins were enriched in signal transduction and endocrine-related pathways, including the calcium signaling pathway, oxytocin signaling pathway, cAMP signaling pathway, and insulin secretion (Figure 7B). Consistent with the KEGG pathway enrichment in carbohydrate metabolism, protein domain analysis revealed that Q1 proteins were enriched in carbon metabolism enzyme-related domains, including phosphorylase b kinase C-terminal domain and fructose-bisphosphate aldolase class-I domain, whereas Q2 proteins showed enrichment in malic enzyme N-terminal domain and malic enzyme NAD-binding domain. In contrast, Q3 and Q4 proteins were enriched in cytoskeletal-related domains, including the LIM domain (Figure 7C). These findings suggested that the functions of DAPs are tightly coupled to their expression patterns.

### 3.4. Protein–Protein Interaction (PPI) Network Analysis of Proteins

Furthermore, we constructed PPI networks for the DAPs in Cytoscape using the STRING database, including only interactions with a combined score > 0.8. The results contained three main clusters comprising 68 proteins (Figure 8). One cluster was mainly involved in glycolysis/gluconeogenesis-related enzymes, including GAPDH, TPI1, PGM1, PGAM1, GPI, PFKM, PFKL, ALDOC, LDHA, and FBP2. The second cluster encompasses proteins involved in purine metabolism, such as HPRT1, NT5E, NT5C2, DGUOK, GMPR2, AMPD1, and ADSS1. The third cluster involved proteins associated with muscle contraction (RYR2, CAMK2A, PPP1R12A, PPP1R12B, PPP1CC, PPP1CA, and PPP1R3A), and protein homeostasis (EIF2S1, EIF4A2, EIF2S3, PSMD14, and MRPS6). The coordinated changes in the abundance of these hub proteins may be associated with intermuscular functional differences, which could ultimately influence muscle quality.

## 4. Discussion

Intrinsic muscle properties, encompassing color, tenderness, juiciness, flavor, and nutritional value, are core meat quality attributes that drive consumer acceptance and market value [2,24]. Prior research indicates that these properties are governed by factors such as genetics, age, sex, and anatomical location, which collectively determine the structure and composition of skeletal muscle [3,25,26]. However, the relative contributions of these factors to meat quality remain unclear. Here, we assessed meat quality traits, including meat color, pH, shear force, water loss rate, and AA, IMF, and FA contents, in PEM and LM of chickens in age-, breed-, and sex-specific comparisons. Our results showed that LM (rich in slow-twitch fibers) had significantly higher pH, shear force, and WHC than PEM (composed predominantly of fast-twitch fibers) in all age, breed, and sex groups examined. These findings are consistent with previous studies showing higher pH and WHC in chicken LM than in PEM across different slaughter ages or breeds [27,28].

Meat quality traits are not isolated events [29,30]. The ultimate pH of meat is an integrative outcome of postmortem metabolism, with downstream effects on the WHC, color, and tenderness [6,30]. Muscles with high glycolytic potential often show a faster early pH decline and lower ultimate pH, whereas oxidative muscles commonly exhibit a more gradual pH decline and higher ultimate pH. When the ultimate pH of meat falls below 5.4, close to the average isoelectric point (pI) of muscle proteins, WHC is minimal [6]. In our study, the ultimate pH values of PEM in AAB and 817C averaged below 6.0. Tenderness is the most important eating quality attribute of meat. Muscle connective tissue content and solubility greatly influence meat texture and are thus critical determinants of meat tenderness and palatability [6]. Chen et al. [31] reported that regardless of chicken breed, LM had significantly higher total soluble collagen content and shear force values than PEM. In our study, the shear force values of both PEM and LM increased progressively with age before AAB reached market age, with LM exhibiting significantly higher shear force than PEM at all time points examined. Notably, this fiber-type-related pattern was reversed in cooked chicken meat [28,32], suggesting that WHC may be a key factor influencing shear force in cooked meat. In chickens, LM generally exhibits a darker color than PEM due to its higher myoglobin content [3,6]. In our study, although L*, a*, and b* values did not differ significantly between the two muscle types at market age (42 d for AAB, 56 d for 817C) in either breed, all three parameters were higher in LM than in PEM of AAB during early growth. This disparity progressively diminished because L* and b* increased in PEM but decreased in LM during early growth of AAB.

Wenchang chickens (WCC), a representative local yellow-feathered broiler breed in China, have a large consumer market due to their excellent flavor [33]. Our results showed that WCC differed significantly from the other two broiler breeds in protein and IMF contents in both PEM and LM. Due to the substantial difference in market age between WCC and the other two breeds, these between-breed differences may be jointly influenced by breed and age. However, the intermuscular differences between PEM and LM in protein and IMF content, AA composition, and FA profiles remained consistent across all three breeds. Specifically, LM had significantly higher IMF content, proportions of umami and aromatic AAs, and absolute and relative total UFA levels than PEM, but substantially lower protein content and proportions of bitter AAs. These results are consistent with previous studies showing that IMF content was lower in PEM than in LM, whereas protein content showed the opposite trend in the breeds and ages examined [27,31,34]. The relative composition of muscle fiber types determines the overall postmortem muscle metabolism and, ultimately, the fresh meat quality [6]. The substantial differences in meat quality between PEM and LM observed in this study may be related to their distinct myofiber-type compositions, which drive metabolic and structural variation.

In meat science, proteomics is a promising tool for understanding meat quality formation and identifying biomarkers by analyzing postmortem muscle protein interactions [35,36,37]. However, major challenges remain in characterizing meat quality and in ensuring accuracy, stability, and industrial applicability of meat quality prediction [38]. Certain consistent pathways were reported in association with beef tenderness or pH across studies, while the concordance of putative biomarkers remains generally poor [35,36,39]. A fiber-centered framework for dissecting meat quality variation may help anticipate which molecular events explain most quality variation in a given muscle and context [30]. In this study, using deep proteomic analysis, we identified 4413 proteins in two representative muscle types (PEM and MGM). PCA showed robust separation between the two groups, and 516 DAPs were identified. Functional enrichment analysis of protein domains, GO, and KEGG pathways for these DAPs indicated that fiber type-related functional differences in chickens mainly involve carbohydrate metabolism, purine metabolism, calcium signaling, and proteostasis. Accordingly, multiple enzymes in glycolysis/gluconeogenesis (GAPDH, TPI1, ENO3, PGM1, PGAM1, GPI, PFKM, PFKL, ALDOC, LDHA, and FBP2) were differentially abundant between the two muscle types, with consistently higher levels in fast-twitch PEM. Accumulating evidence indicates that GAPDH and TPI1 affect meat tenderness, color, and WHC, supporting their potential as meat quality biomarkers [38]. PGM1, a vital glycolytic enzyme providing glucose-6-phosphate (G6P) for ATP production from glycogen in living and early postmortem muscles, is a well-known biomarker for poultry meat quality [40]. Notably, LDHA and ENO3 expression, associated with chicken muscle fiber types, is also closely related to PEM growth and development in chicken [41]. Although ENO3 abundance varies among lamb muscle types, its expression correlates strongly with meat quality, indicating that ENO3 is a robust biomarker for lamb meat quality [42,43]. In cattle, although FHL1 expression did not differ significantly among five bovine muscle types, it was strongly negatively correlated with TPI1 [44]. FHL1 is a robust biomarker of beef quality, and its content correlated negatively with tenderness and L* and positively with IMF and a* [45,46]. In chicken wooden breast, Zhang et al. [47] found that FHL1 was more abundant in PEM and proposed that it may affect shear force by regulating muscle fiber hypertrophy and assembly. In our study, FHL1 was significantly enriched in the LIM domain and highly abundant in slow-twitch MGM. These findings suggested that FHL1 may be associated with the higher shear force in MGM, although further validation is needed.

In our study, purine metabolism-related proteins formed the second functional cluster in the PPI analysis. Purine nucleotide cycle-related enzymes (e.g., AMPD1 and ADSS1) were more abundant in fast-twitch PEM. In contrast, enzymes involved in purine salvage and nucleoside recycling (e.g., HPRT1, NT5E, NT5C2, GMPR2, and DGUOK) were more abundant in slow-twitch MGM. This differential expression pattern suggests marked differences in purine metabolism between the two muscles. Lu et al. [48] reported similar muscle-specific differences in Pingliang Red cattle using integrated proteomic and metabolomic data. These differences may reflect distinct energy metabolic strategies. Fast-twitch muscles rely on a highly active purine nucleotide cycle to rapidly turn over nucleotides and buffer AMP accumulation. In contrast, oxidative slow-twitch muscles rely on efficient salvage and recycling to maintain purine pool stability and mitochondrial function. Notably, NT5E has been reported to participate in nicotinate/nicotinamide metabolism and contribute to nicotinamide riboside (NR) production [49]. In pigs and sheep, the nicotinate/nicotinamide metabolism pathway has been implicated in meat color regulation [50,51]. Zhu et al. [52] reported that NR-supplemented pork showed greater color stability during retail display, which may be attributable to the ability of NR to preserve mitochondrial integrity and attenuate apoptosis activation in the early postmortem period. Whether the higher NT5E abundance in chicken muscle contributes to meat color variation requires further investigation.

The ryanodine receptor (RyR) family comprises intracellular Ca^2+^ release channels located on the sarcoplasmic/endoplasmic reticulum (SR/ER) membrane that release stored Ca^2+^ into the cytosol and regulate muscle contraction [53]. After slaughter, stored Ca^2+^ can be continuously released into the cytosol via RYR2, promoting actomyosin cross-bridge formation and rigor mortis, while activating Ca^2+^-dependent proteases [54]. Elevated RYR2 expression in yak longissimus thoracis muscle was potentially associated with poorer postmortem tenderness, possibly due to a more severe postmortem imbalance in calcium homeostasis that may affect proteolysis and tenderness development [54]. In our study, RYR2 was more abundant in slow-twitch MGM. Although direct evidence regarding the role of RYR2 in chicken meat quality remains lacking, multiple genomic selection studies have identified *RYR2* as a selected gene in Chinese yellow-feathered chickens [55,56,57]. These studies suggest that RYR2 may be a candidate gene involved in selection for chicken meat quality. Furthermore, we found that translation initiation factors (EIF2S1, EIF2S3, and EIF4A2) were less abundant, whereas deubiquitinase PSMD14 and mitochondrial ribosomal protein MRPS6 were more abundant in slow-twitch MGM during the early postmortem period. These differences may reflect intermuscular variation in the capacity for cytosolic translation and mitochondrial protein synthesis, which could potentially affect meat quality by influencing postmortem energy metabolism and myofibrillar protein degradation. However, these potential mechanisms still warrant further experimental validation.

## 5. Conclusions

In this study, we evaluated variation in chicken meat quality with respect to age, breed, sex, and muscle location. Marked differences in meat quality traits (pH, meat color, tenderness, WHC, amino acid composition, intramuscular fat composition, and intramuscular fat content) between PEM and LM were observed in the age, sex, and breed comparisons. High-throughput quantitative proteomic analysis revealed that intermuscular functional differences were mainly involved in carbohydrate metabolism, purine metabolism, calcium signaling, and proteostasis. Proteins identified by PPI analysis, including GAPDH, TPI1, PGM1, LDHA, ENO3, FHL1, NT5E, RYR2, and MRPS6, may be associated with intermuscular meat quality variation in the early postmortem period. However, further validation is needed to confirm their potential as protein biomarkers. These findings advance our understanding of the molecular basis of chicken meat quality and may facilitate postmortem quality control in the poultry meat industry.

## Figures and Tables

**Figure 1:**
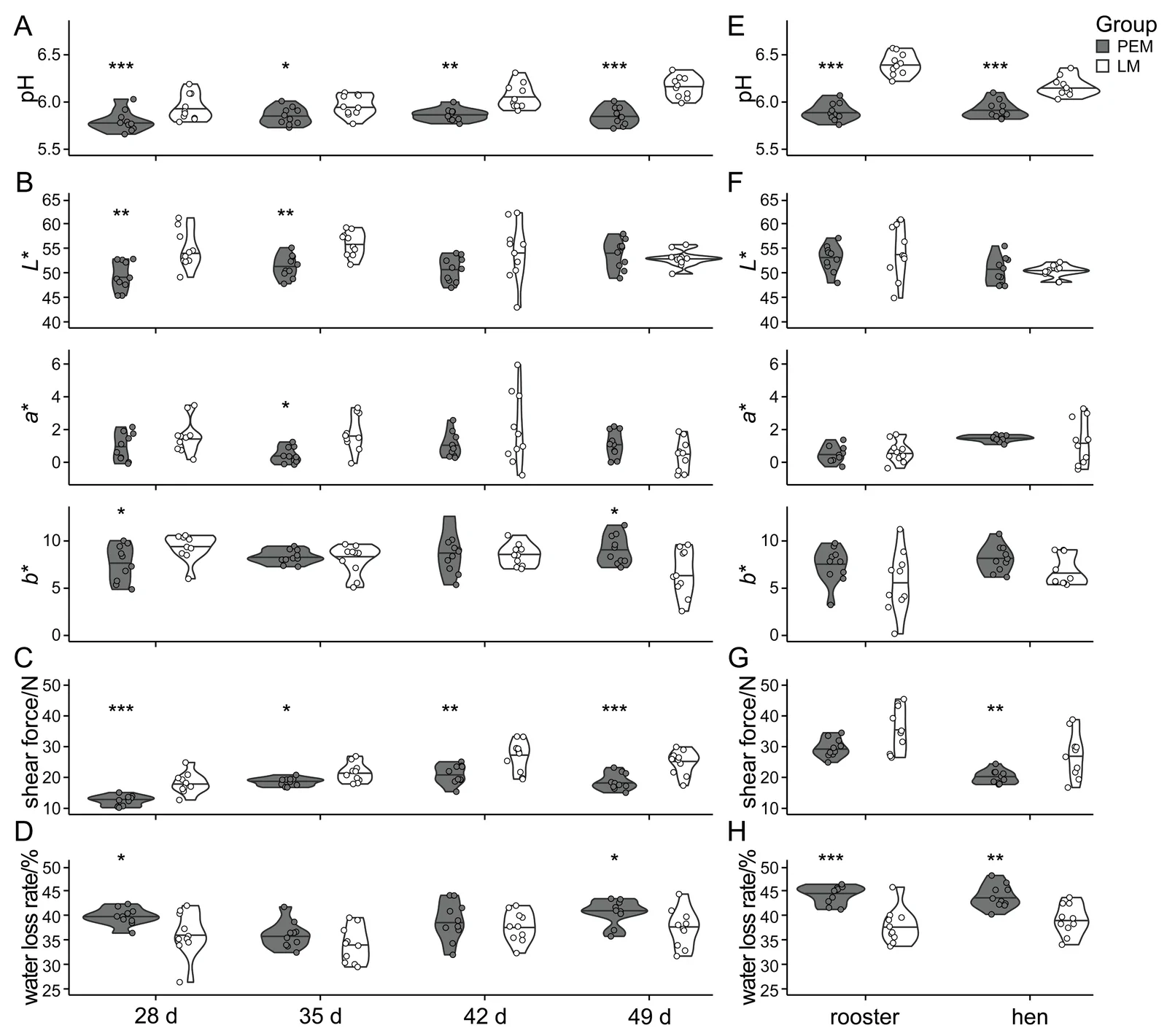
Difference in meat quality parameters (pH, meat color, shear force, and water loss rate) between PEM and LM. Violin plots show the distribution of individual data points (*n* = 10 per group). The horizontal line indicates the mean. (**A**–**D**) Changes in meat quality between PEM and LM of AAB from 28 to 49 d. (**E**–**H**) Sex-related differences in meat quality between PEM and LM of 817C. Asterisks indicate significant differences between groups (*: *p* < 0.05; **: *p* < 0.01; ***: *p* < 0.001).

**Figure 2:**
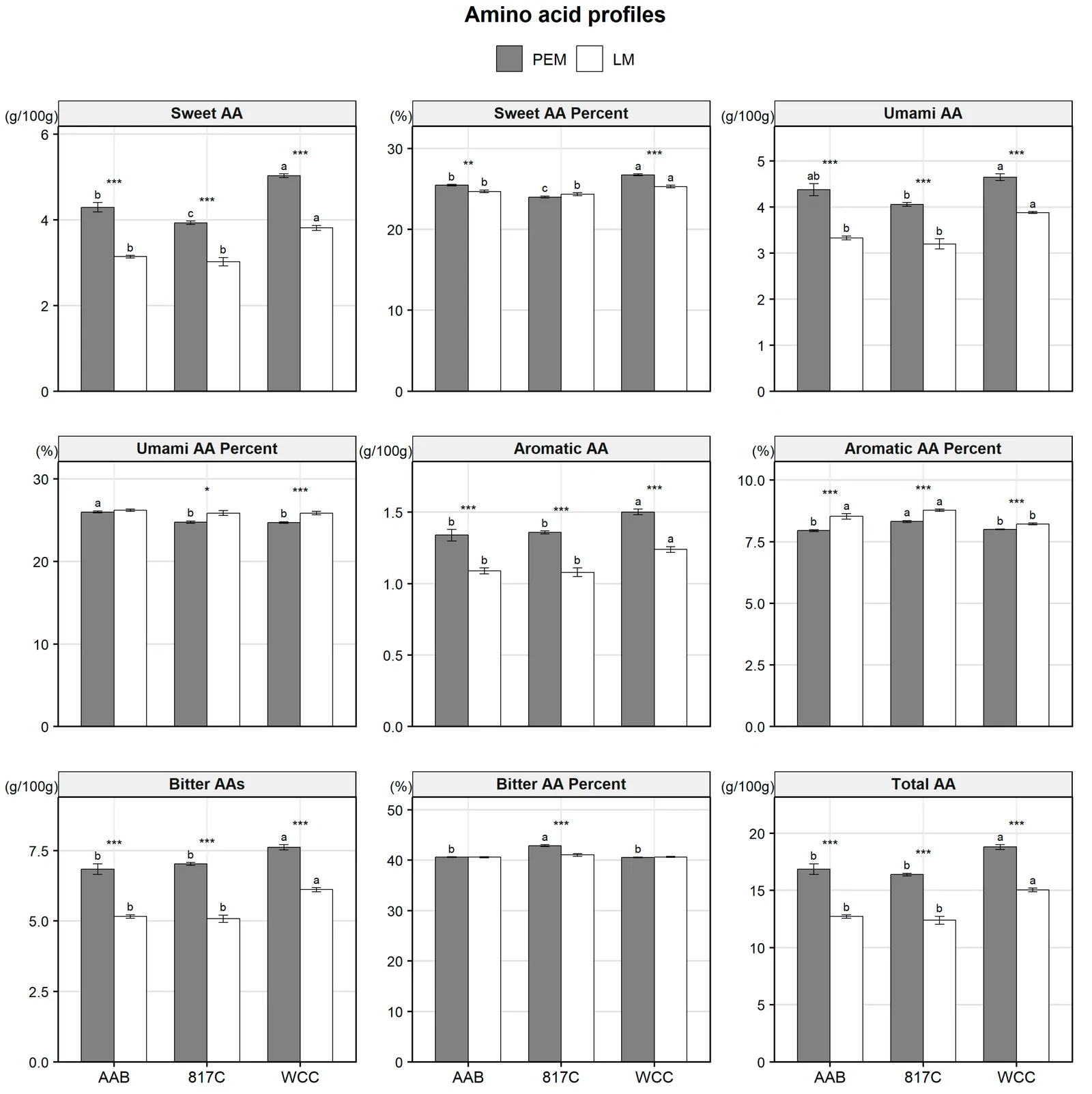
Amino acid (AA) profiles in the PEM and LM of three chicken breeds at market age. Data are mean ± SEM (AAB, *n* = 10; 817C, *n* = 9; WCC, *n* = 15). Amino acids were grouped as sweet AAs (Gly, Ala, Ser, Thr, Pro, and Met), umami AAs (Glu and Asp), aromatic AAs (Tyr and Phe), and bitter AAs (Lys, Arg, His, Leu, Ile, and Val). Within-breed comparisons (PEM vs. LM) were performed using paired-samples *t*-tests (*: *p* < 0.05; **: *p* < 0.01; ***: *p* < 0.001). Between-breed comparisons (among three breeds within the same muscle) were performed using one-way ANOVA followed by Tukey’s HSD test; different lowercase letters indicate *p* < 0.05.

**Figure 3:**
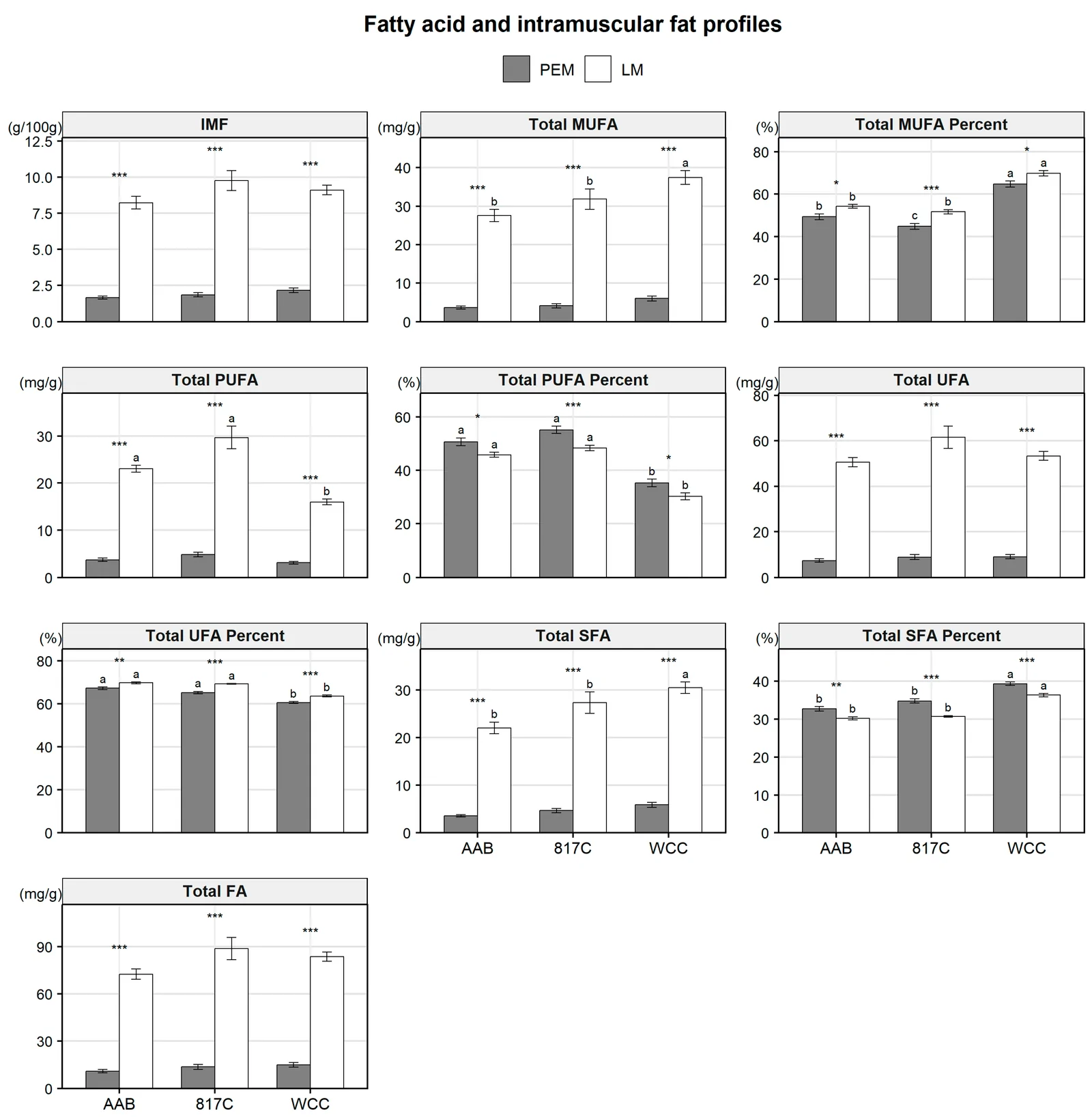
Intramuscular fat (IMF) and fatty acid (FA) profiles in the PEM and LM of three chicken breeds at market age. Data are the mean ± SEM (AAB, *n* = 10; 817C, *n* = 9; WCC, *n* = 15). Within-breed comparisons (PEM vs. LM) were performed using paired-samples *t*-tests (*: *p* < 0.05; **: *p* < 0.01; ***: *p* < 0.001). Between-breed comparisons (among three breeds within the same muscle) were performed using one-way ANOVA followed by Tukey’s HSD test; different lowercase letters indicate *p* < 0.05.

**Figure 4:**
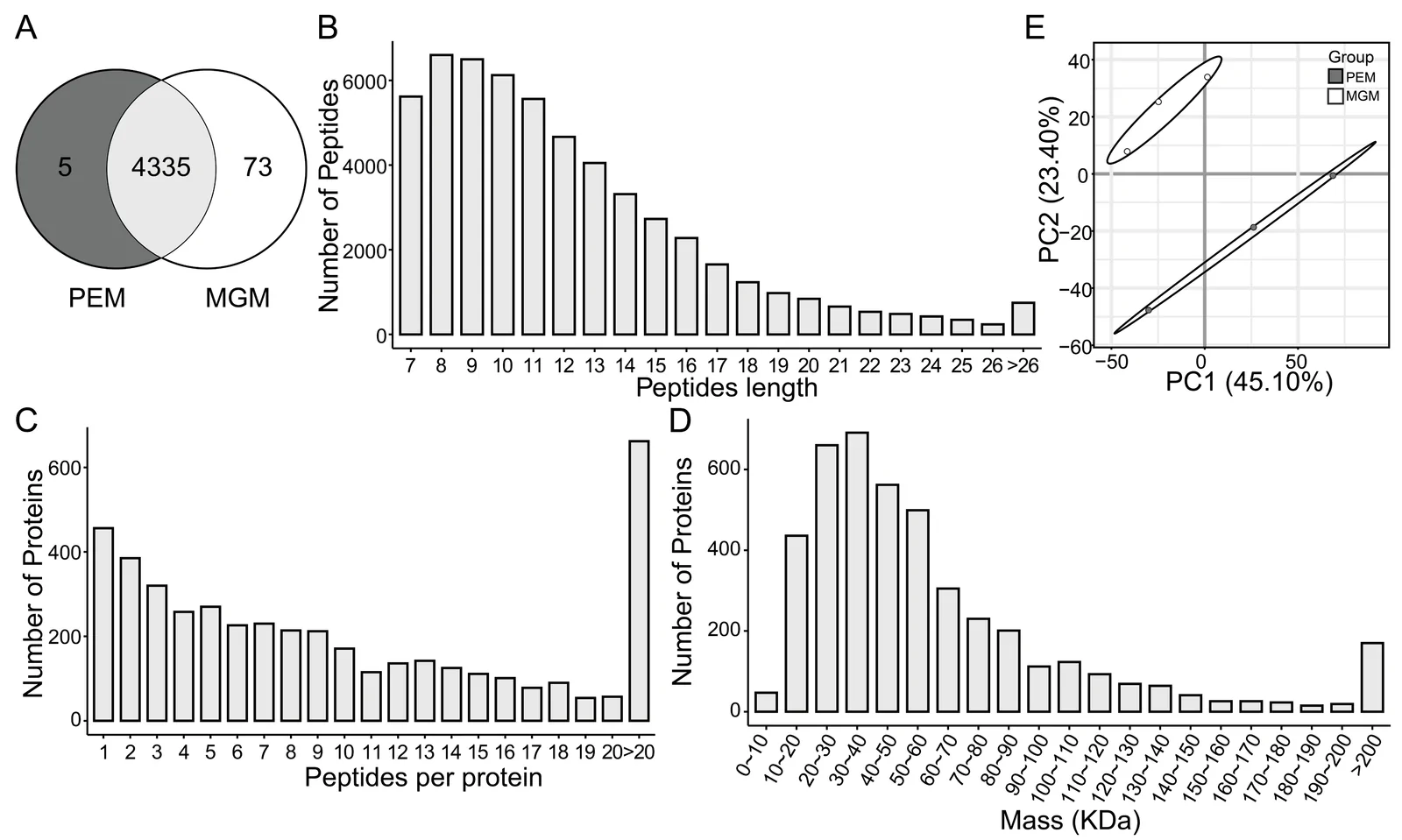
Overview of proteomic analysis of PEM and MGM. (**A**) Venn diagram of proteins between PEM and MGM. (**B**) Distribution of peptide lengths. (**C**) Distribution of peptide counts. (**D**) Distribution of protein molecular weights. (**E**) PCA of identified proteins. The ellipses indicate the 95% confidence intervals for each group.

**Figure 5:**
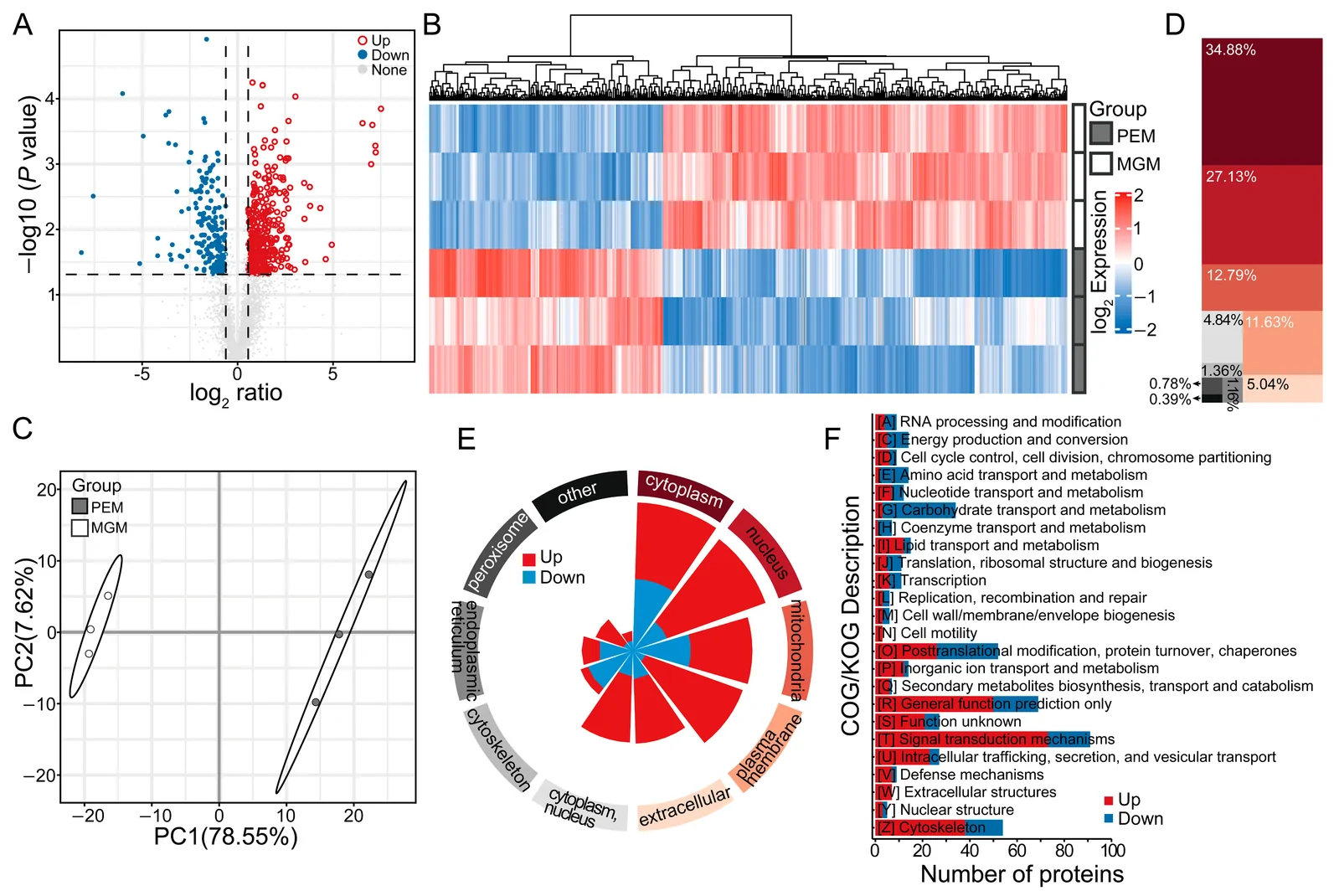
Differentially abundant proteins (DAPs) between PEM and MGM. (**A**) Volcano plot of DAPs. (**B**) Hierarchical clustering heatmap of DAPs. (**C**) PCA of DAPs. (**D**) Subcellular distribution of DAPs. (**E**) Comparative subcellular distribution of more abundant and less abundant DAPs. (**F**) KOG categories of more abundant and less abundant DAPs. Red/blue colors denote up/down-regulated proteins, dashed lines in (**A**) are significance thresholds, and ellipses in (**C**) are 95% confidence intervals.

**Figure 6:**
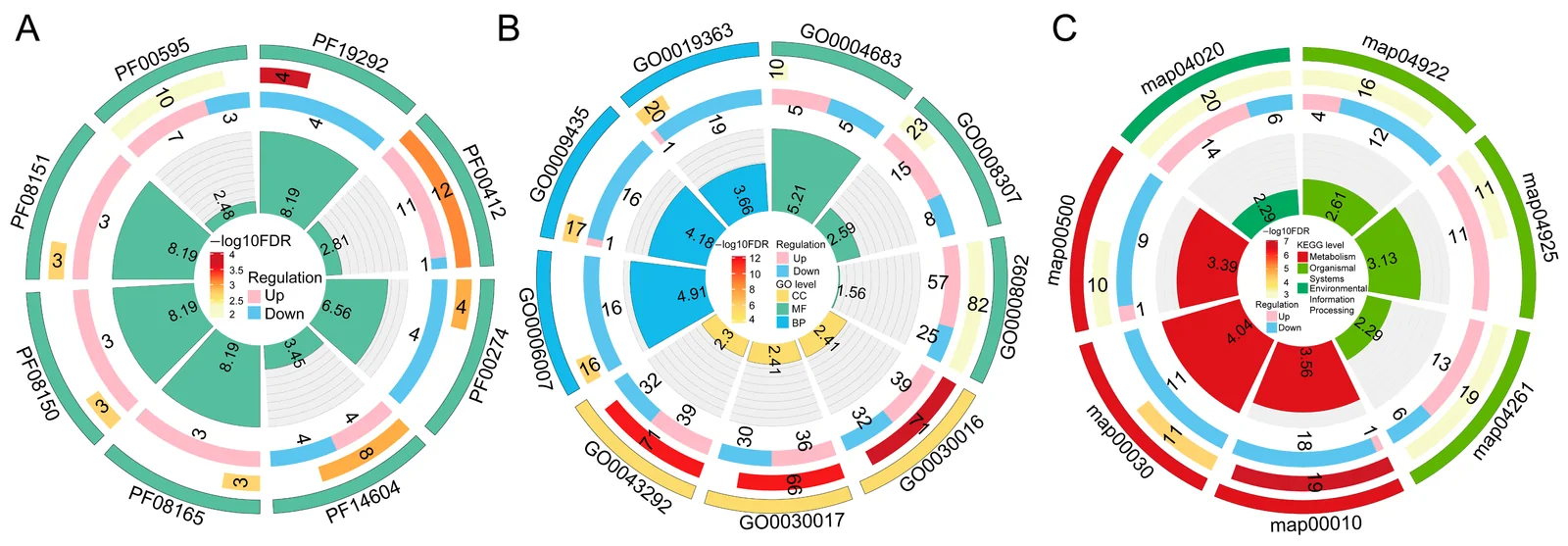
Functional analyses of DAPs identified between PEM and MGM. (**A**) Protein domain enrichment. (**B**) GO enrichment. (**C**) KEGG pathway enrichment.

**Figure 7:**
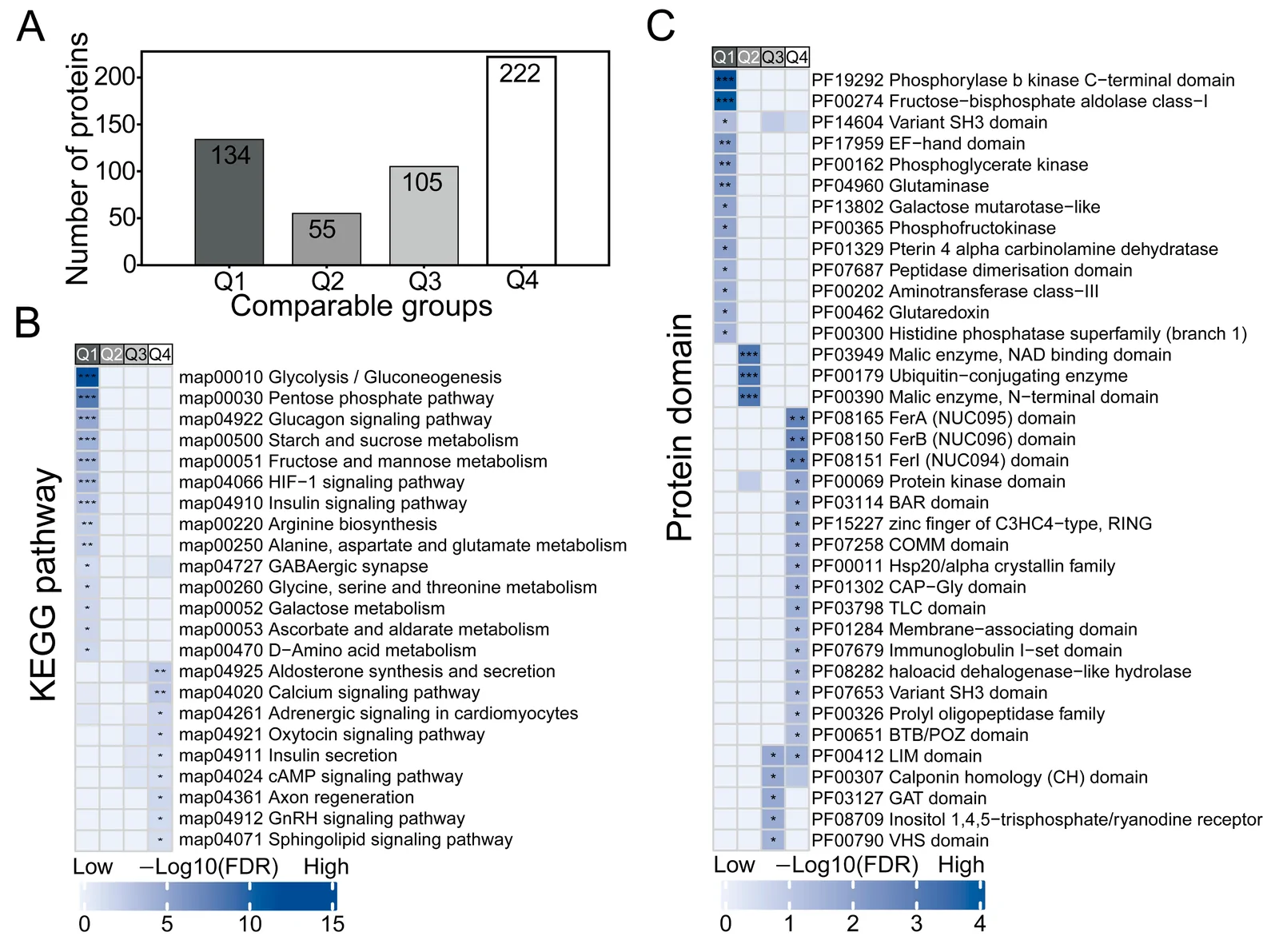
Clustering analyses of DAPs identified between PEM and MGM. (**A**) Grouping of DAPs by fold change (FC) between PEM and MGM. Q1, strongly less abundant (FC < 0.5); Q2, mildly less abundant (0.5 ≤ FC < 0.667); Q3, moderately more abundant (1.5 < FC ≤ 2.0); Q4, strongly more abundant (FC > 2.0). (**B**) KEGG pathway enrichment clustering of DAPs. (**C**) Protein domain enrichment clustering of DAPs. Asterisks indicate significant differences between groups (*: *q* < 0.05; **: *q* < 0.01; ***: *q* < 0.001).

**Figure 8:**
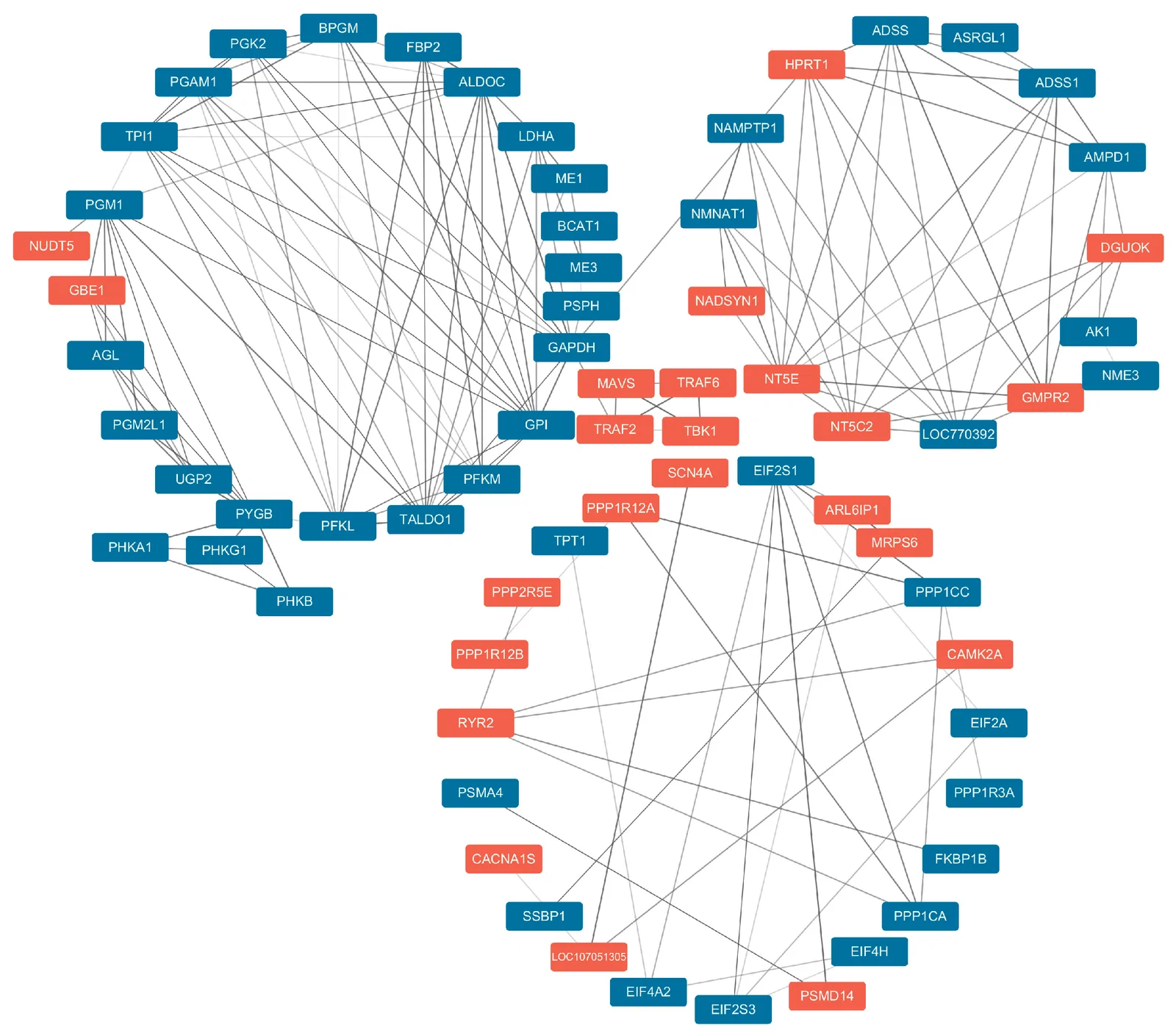
PPI network of DAPs identified between PEM and MGM. Proteins more abundant in MGM are shown in red, whereas less abundant proteins are shown in blue. The thickness of each line is proportional to the combined score.

**Table 1:** Summary of experimental design and animal sampling.

Experiment	Breed	Age	Sex	Muscle Samples
Age effect on edible meat quality	AAB	28, 35, 42, 49 d	Male	PEM, LM
Sex effect on edible meat quality	817C	56 d	Male and female	PEM, LM
Breed effect on meat nutritional quality	AAB, 817C, WCC	Market age: 42 d (AAB), 56 d (817C), 120 d (WCC)	Male	PEM, LM
Quantitative proteomics	WCC	120 d	Male	PEM, MGM

**Table 2:** Number of proteins and unique peptides identified in PEM and MGM.

Term	Number
Identified peptides	55,552
Unique peptides	22,223
Identified proteins	4413
Comparable proteins	4335

## Data Availability

The mass spectrometry proteomics data have been deposited in the ProteomeXchange Consortium via the PRIDE partner repository with the dataset identifier PXD085713. The remaining data presented in this study are included in the article and/or Supplementary Materials. Further inquiries can be directed to the corresponding author.

## Supplementary Materials

The following supporting information can be downloaded at https://www.mdpi.com/article/10.3390/foods15214010/s1, Table S1: A total of 516 DAPs were identified between PEM and MGM. Table S2: Protein domain enrichment analysis of DAPs identified between PEM and MGM. Table S3: GO enrichment analysis of DAPs identified between PEM and MGM. Table S4: KEGG pathway enrichment analysis of DAPs identified between PEM and MGM.
